# Clinical predictors associated with prolonged pneumoperitoneum time in laparoscopic living donor nephrectomy

**DOI:** 10.1007/s10157-025-02663-2

**Published:** 2025-03-26

**Authors:** Hiroki Kato, Keita Nakane, Ayaka Okamoto, Teppei Nishiwaki, Kojiro Niwa, Masayuki Tomioka, Tomoki Taniguchi, Makoto Kawase, Kota Kawase, Koji Iinuma, Yuki Tobisawa, Takuya Koie

**Affiliations:** Department of Urology, Gifu University Graduate School of Medicine, 1-1 Yanagito, Gifu, 501-1194 Japan

**Keywords:** Kidney transplantation, Living donor, Laparoscopic donor nephrectomy, Pneumoperitoneum time

## Abstract

**Background:**

Kidney transplantation (KT) is a useful treatment option for patients with end-stage chronic kidney disease to avoid dialysis and achieve a good quality of life. In Japan, approximately 90% of kidneys for KT are obtained from living kidney donors. Laparoscopic renal nephrectomy (LDN) is the most commonly performed KT procedure in Japan. We aimed to determine the clinical variables that influence the prolongation of pneumoperitoneum time (PT) in LDN.

**Method:**

This retrospective study was carried out on 218 consecutive patients who underwent LDN at Gifu University Hospital. T The enrolled patients were divided into two groups according to the third quartile of PT in the LDN, with those in the lower third quartile (Q3) as Group 1 and those in the upper Q3 as Group 2. The primary endpoint was identification of predictive factors for prolonged PT.

**Result:**

In total, 178 patients were included in the analysis. For all patients, the median PT, estimated blood loss, and warm ischemic time were 170 min, 20 mL, and 4 min, respectively. Significantly longer PT was observed in Group 2 than in Group 1. Multiple regression analysis results showed that male donors, body mass index ≥ 25 kg/m^2^, and donors with more than two renal arteries were independent predictive factors for PT prolongation.

**Conclusion:**

Male sex, BMI ≥ 25 kg/m^2^, and two or more renal arteries are predictive factors for prolonged PT for donors in LDN.

## Introduction

Kidney transplantation (KT) is recognized as a useful treatment option for patients with end-stage chronic kidney disease to avoid dialysis and achieve a good quality of life [1]. In an epidemiological study of KT in 2022, only approximately 30% of KT in the United States were performed with kidneys from living kidney donors compared with cadaveric kidney donors, whereas in Japan, approximately 90% of donated kidneys were obtained from living kidney donors [2, 3]. Although various minimally invasive surgical techniques are used for donor nephrectomy (DN), laparoscopic renal nephrectomy (LDN) is the most commonly used in Japan [4–6]. In living KT, surgery is usually performed simultaneously on the donor and recipient; however, prolonged DN may increase the risk of perioperative complications in the recipient [7]. Although DN should therefore be performed as quickly and safely as possible, not only the skill of the surgeon and the surgical procedure, but also the status of the donor has been suggested to affect DN time [8]. We aimed to determine the clinical variables that influence the prolongation of pneumoperitoneum time (PT) in LDN.

## Patients and methods

### Patients

We conducted this study with the approval of the Institutional Review Board at Gifu University (Approval No. 2021-B129). Given the retrospective nature of this study, informed consent was not obtained individually from the enrolled patients; instead, consent was obtained by opt-out. Ethical guidelines and regulations in Japan stipulate that written consent is not required for retrospective cohort studies that use existing records if the research information is publicly available.

This retrospective study was carried out on 218 consecutive patients who underwent LDN at the Gifu University Hospital between February 2011 and January 2023. Twelve patients who underwent open DN, 26 who underwent transperitoneal LDN, and two who underwent hand-assisted LDN were excluded. The following clinical parameters of the donors were retrospectively extracted: age, sex, height, weight, body mass index (BMI), preoperative serum creatinine level, smoking history, presence of diabetes and hypertension, harvested kidney location, and number of renal arteries. Data on the following factors related to KT were collected: PT, estimated blood loss (EBL), warm ischemia time (WIT), and incidence of perioperative complications (Table 1). WIT was defined as the time from the clamping of the renal artery to the start of Euro-Collins solution perfusion into the harvested kidney.

**Table 1:** Perioperative results

Variables	Group 1 (N = 134)	Group 2 (N = 44)	P value
Intraoperative complication (number, %)	2 (1.5)	0 (0)	> 0.999
EBL (mL, median, IQR)	20 (10–35)	30 (20–80)	0.721
WIT (minute, median, IQR)	4 (3–5)	4 (4–6)	0.004
Open conversion rate (number, %)	1 (0.7)	0 (0)	> 0.999

*Q* Quartile, *IQR* Interquartile range, *EBL* Estimated blood loss, *WIT* Warm ischemic time

t-test、Pearson ‘s Chi-squared test

### Surgical procedures

LDN was performed in the lateral decubitus position under general and epidural anesthesia. The port position for left LDN was a 12 mm trocar at the inferior margin of the 12th rib above the posterior axillary line, a 12 mm camera port at the midpoint between the inferior margin of the 11th rib and the iliac crest above the midaxillary line, and a 5 mm trocar outside the four lateral fingers of the camera port. In right LDN, a 5 mm port was placed at the inferior margin of the 12th rib above the posterior axillary line, a 12 mm port was placed four fingers lateral to the camera port, and the camera port was placed in the same position as in left LDN on the right midaxillary line. The pneumoperitoneal pressure was 8–10 mmHg during LDN in all patients. Immediately prior to processing the renal vessels, 100 mL of mannitol and 3000 units of heparin were administered intravenously. The renal artery and vein were cut using an EndoGIA™ Ultra (Medtronic, Minneapolis, MI, USA). An 8 cm Pfannenstiel incision was made above the pubis and an Alexis O Wound Protector^®^ (Applied Medical, Rancho Santa Margarita, CA, USA) was placed. The kidney used for KT was removed using an EndoCatch^™^ II (Medtronic, Minneapolis, MN, USA) and immediately perfused from the renal artery with cooled Euro-Collins solution, after which KT was performed.

The kidneys to be harvested were selected based on the number of renal arteries and preoperative renal scintigraphy with 99mTc-DTPA, in which the renal function was at least 10% lower.

### Statistical analysis

The enrolled patients were divided into two groups according to the third quartile of PT in the LDN, with those in the lower third quartile (Q3) as Group 1 and those in the upper Q3 as Group 2. The primary endpoint was identification of predictive factors for prolonged PT. All statistical evaluations were performed using EZR (Saitama Medical Center, Jichi Medical University, Saitama, Japan), a graphical user interface for R (The R Foundation for Statistical Computing, Vienna, Austria) [9]. A t-test or Pearson's chi-square test was employed to compare various parameters between the two groups. Logistic regression analysis was employed to investigate variables that contribute to a PT ≥ Q3. Multiple regression analysis was also performed to examine the preoperative and operative factors that may predict PT prolongation. Statistical significance for all comparisons was set at a two-sided P-value < 0.05.

## Results

A total of 178 patients were included in this analysis. Table 2 shows the preoperative covariates of the two groups. The median age was 61 years (interquartile range [IQR], 52–67 years), the sex was predominantly female (60.1%), and 45 patients (25.2%) had a BMI of ≥ 25 kg/m^2^. Preoperative donor comorbidities included diabetes mellitus in 10 patients (5.6%) and hypertension in 38 (21.3%). More than two renal arteries were found in 31 patients (16.9%), and the right kidney was harvested in 19 patients (10.7%).

**Table 2:** Comparison of preoperative factors in enrolled patients

Variables	Group 1 (N = 134)	Group 2 (N = 44)	P value
Age (year, median, IQR))	61 (52–67)	61 (53–67)	0.889
Sex (number, %)			
Male	92 (68.7)	15 (34.1)	< 0.001
Female	42 (31.3)	29 (65.9)	
Body mass index (kg/m^2^, median, IQR)	22.2 (20.0–24.5)	23.3 (22.0–26.3)	0.006
Smoking history (number, %)	18 (13.4)	15 (34.1)	0.005
Diabetes mellitus (number, %)	8 (5.9)	2 (4.5)	> 0.999
Hypertension (number, %)	25 (18.6)	13 (29.5)	0.223
Preoperative serum Cr (mg/dL, median, IQR)	0.64 (0.58–0.73)	0.73 (0.61–0.83)	0.011
Two or more renal arteries (number, %)	17 (12.7)	13 (29.5)	0.020
Right nephrectomy (number, %)	14 (10.4)	5 (11.4)	> 0.999

*Q* Quartile, *IQR* Interquartile range, *CR* Creatinine

t-test、Pearson ‘s Chi-squared test

**Fig. 1:**
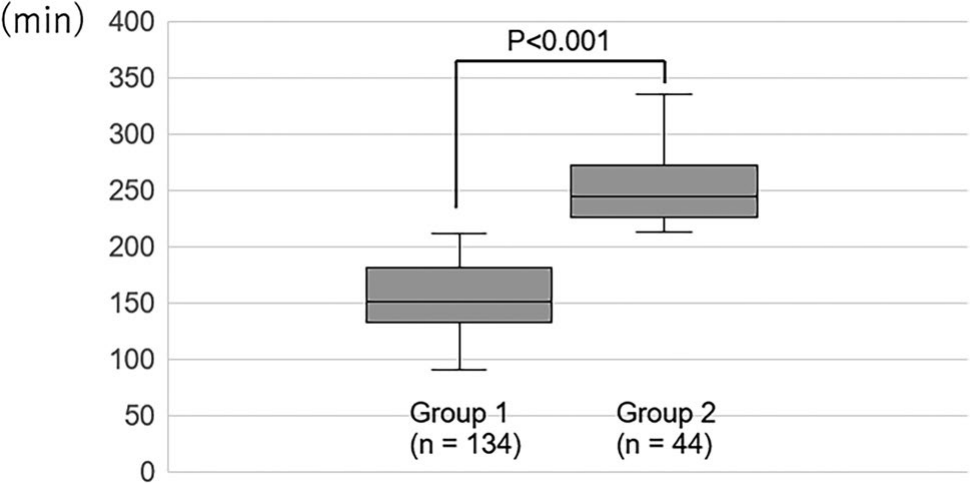
Significantly longer PT was observed in the patients in quartile ≥ 3 than in those in quartile ≤ 2 (p < 0.001)

For all patients, the median PT, EBL, and WIT were 170 min (IQR, 138–213 min), 20 mL (IQR, 10–40 mL), and 4 min (IQR, 3–5 min), respectively. Perioperative complications were observed only in Group 1: one patient developed postoperative pseudogout and one required conversion from laparoscopic to open surgery due to intraoperative vascular injury. Figure 1 shows the PT values of the two groups. Group 2 had significantly longer PT than Group 1 (Fig. 1). Median pneumoperitoneum time and total operative time for Group1 and Group2 were 151.5 min (IQR, 133–181), 183 min (IQR, 161.2–210.5) and 248.5 min (IQR, 229.75–271), 280 min (IQR, 262–301), respectively (p < 0.001).

Moreover, we obtained data on the Brinkmann index from patients in whom the number of cigarettes smoked per day and the number of years smoked were noted in their medical records and analyzed the relationship with pneumoperitoneum time. The Pearson's correlation coefficient was 0.161 (95% CI: −0.26 to 0.53) and p-value was 0.453. We found no correlation between the Brinkmann index and pneumoperitoneum time.

The covariates predicting PT prolongation were examined using multiple regression analysis (Table 3). The adjusted coefficient of determination (R^2^) was 0.193. The results showed that male sex, BMI ≥ 25 kg/m^2^, and more than two renal arteries were independent predictive factors for PT prolongation. PT was approximately 23 min longer in male donors than in females, approximately 18 min longer in donors with BMI ≥ 25 kg/m^2^ than in those with BMI < 25 kg/m^2^, and approximately 34 min longer in donors with two or more renal arteries than in those with one renal artery. Subsequently, logistic regression analysis was performed with pre- and postoperative covariates to search for independent predictors of PT ≥ Q3 (Table 4). On multivariate analysis, male donors and those with more than one renal artery were statistically significant predictors of PT ≥ Q3.

**Table 3:** Multiple regression analysis to predict PT

Variables	B	95% CI	P value
Intercept	119.64	96.55—142.73	
Age			
≥ 65	−3.03	−4.85 – 35.31	0.698
< 65	ref		
Sex			
Male	23.12	6.88 – 39.37	0.006
Female	ref		
BMI			
≥ 25	17.49	0.05—34.94	0.049
< 25	ref		
Smoking history			
Yes	15.23	−4.85—35.31	0.136
No	ref		
DM			
Yes	−15.94	−47.40 – 15.51	0.318
No	ref		
HTN			
Yes	5.45	−13.60 – 24.51	0.573
No	ref		
Renal artery			
≥ 2	34.13	15.09—53.18	< 0.001
1	ref		
LDN			
Right	14.18	−9.63—38.00	0.241
Left	ref		

*B* Regression coefficient, *CI* Confidence interval, *BMI* Body mass index, *DM* Diabetes mellitus, *HTN* Hypertension, *LDN* Laparoscopic donor nephrectomy

**Table 4:** Logistic regression analysis to predict PT over Q3

	Univariate analysis			Multivariate analysis		
Variables	OR	95% CI	P value	OR	95% CI	P value
Age						
≥ 65	0.77	0.368 – 1.610	0.366	0.68	0.290 – 1.580	0.366
< 65	ref			ref		
Sex						
Male	3.93	1.930 – 8.000	< 0.001	2.74	1.200 – 6.260	0.016
Female	ref			ref		
BMI						
≥ 25	2.28	0.176 – 3,540	0.028	1.51	0.646 – 3.540	0.340
< 25	ref			ref		
Smoking history						
Yes	3.28	1.480 – 7.270	0.004	1.90	0.743 – 4.830	0.181
No	ref			ref		
DM						
Yes	0.727	0.149 – 3.560	0.694	0.963	0.175 – 5.300	0.965
No	ref			ref		
Hypertension						
Yes	1.75	0.806 – 3.820	0.156	1.26	0.489 – 3.270	0.629
No	ref			ref		
Renal artery						
≥ 2	2.77	1.220 – 6.300	0.015	2.68	1.080 – 6.670	0.034
1	ref			ref		
LDN						
Right	1.06	0.360 – 3.140	0.913	1.07	0.312 – 3.650	0.918
Left	ref			ref		

*OD* Odds ratio, *CI* Confidence interval, *BMI* Body mass index, *DM* Diabetes mellitus, *LDN* Laparoscopic donor nephrectomy

## Discussion

KT for patients with end-stage chronic kidney disease is recognized as a useful treatment modality that reduces mortality and improves the quality of life compared with dialysis [1]. In Japan, KT is performed much more frequently with kidneys donated by living donors than by brain- or heart-dead donors [3]. Therefore, minimizing the donor burden in living donor KT is one of the most important issues. LDN has been widely adopted as a less invasive approach than open surgery, and robot-assisted donor kidney harvesting has recently been introduced despite not being covered by insurance in Japan [10–13]. Prolonged operative time for donor nephrectomy has been associated with an increased incidence of postoperative complications after KT, and shortening this time is a critical issue [7]. This study focused on PT, which reflects the operative time for LDN, and examined the possibility of preoperatively predicting PT prolongation by analyzing the correlation between PT and covariates. We found that donors who were male, had a BMI of ≥ 25 kg/m^2^, or had more than two renal arteries been significant predictors of prolonged PT.

In previous studies, male sex was a significant predictor of PT prolongation [14–17]. In a study examining risk factors for prolonged operative time in 700 hand-assisted LDNs over a period of approximately 10 years, the overall mean operative time was 143.7 ± 39.1 min (range, 70–360 min) [14]. Male donors had significantly longer PT than female donors on both univariate (odds ratio [OR] 5.12:95% confidence interval [CI] 3.34–7.98; p < 0.001) and multivariate analysis (OR 2.35:95% CI 1.11–5.03; p = 0.026) [14]. According to a study of 2,477 LDNs, the median operative time in female donors was significantly shorter than that in male donors (72 vs. 75 min, p = 0.001) [15]. Günaydin et al. [16] reviewed 1,864 hand-assisted LDN procedures and reported a mean operative time of 90.5 ± 35.0 min for male donors and 83.7 ± 37.1 min for female donors, with male donors having significantly longer operative time (p < 0.01). The Mayo Adhesive Probability (MAP) score was initially designed to predict the presence of troublesome adherent perirenal fat during partial nephrectomy [18]. In a study using MAP scores to examine risk factors related to operative time for LDN, female donors had lower mean MAP scores than males (0.35 ± 0.86 vs. 1.03 ± 1.29; P < 0.001), and higher MAP scores were significantly associated with longer operative time for LDN [17]. A higher proportion of male donors had elevated MAP scores, which may have contributed to the prolonged PT.

Regarding the eligibility criteria for living kidney donors, UK guidelines recommend careful preoperative evaluation of individuals with a BMI between 30 and 35, and those with a BMI ≥ 35 should be discouraged from donation due to limited data [19]. In contrast, the European Urological Association guidelines make no specific recommendations for donors with a BMI ≥ 30 [20]. Thus, the cutoff BMI value for obese donors remains debatable. A retrospective study of 553 donors undergoing hand-assisted LDN reported significantly longer operative time in donors with BMI ≥ 30 kg/m^2^ than in those with BMI < 30 kg/m^2^, with a difference of only 19 min [21]. Similarly, in a study of 1,741 living donors who underwent LDN, those with a BMI > 28 kg/m^2^ reported a 1.36-fold increase in operative time for LDN compared with those with a BMI ≤ 28 (p = 0.009) [22]. Takagi et al. [22] reported that LDN was 1.38 times more difficult in patients with a BMI > 28 kg/m^2^ than in those with a BMI < 28 (OR: 1.38; 95% CI: 1.08–1.72). A high BMI reflects excess accumulation of body fat, and an increase in perirenal adipose tissue may increase the difficulty of surgery and thus prolong the operative time.

Several studies have reported that LDNs involving multiple renal arteries have longer operative times than those with a single artery [23–25]. In a meta-analysis of 20 studies, the pooled mean operative time was 188 min for LDN with a single artery and 214 min for LDN with two or more arteries [23]. LDNs with two or more arteries showed significantly longer operative time than LDNs with only one artery (mean difference, 15.22 min; 95% CI 8.35–22.09; p < 0.001), with no effect of LDN method [23]. In a study of 1,350 living donors, 288 kidneys (21.3%) had multiple renal arteries [24]. The mean operative time was 71.2 min for LDNs with a single artery and 79.9 min for LDNs with two or more arteries, with a significantly longer operative time for donors with multiple renal arteries (p < 0.001) [24]. Similarly, an examination of 214 KT donors showed that LDNs with a single renal artery had significantly shorter operative time than those with two or more renal arteries (90.3 min vs. 102.1 min; p < 0.001) [25]. In previous reports, as well as in the present study, the operative time for LDNs with two or more renal arteries was significantly longer than that for LDNs with a single artery, approximately 10–15 min [23–25]. The increased operative time for LDN may be attributed to technical difficulties; however, this may not significantly affect the perioperative outcome of LDN [23].

Several limitations are noted in this study. First, the results may be biased because this was a retrospective, single-center cohort study with a relatively small number of cases. Second, the surgical technique was performed exclusively using LDN via the retroperitoneal approach by four relatively experienced surgeons. As LDN was not performed by a single surgeon or using a variety of different approaches, the factors associated with prolonged operative time need further investigation.

In conclusion, this study identified male sex, BMI ≥ 25 kg/m^2^, and two or more renal arteries as predictive factors for prolonged PT in LDN for donors. Preoperative prediction of PT prolongation in LDN may contribute to a shorter operative time and better outcomes in recipients undergoing KT.

## Data Availability

The datasets supporting the conclusions of this study can be made available upon reasonable request to the corresponding author. However, the data are not publicly accessible due to privacy and ethical considerations.
